# Field test results of the motherhood method to measure maternal mortality

**Published:** 2011-01

**Authors:** Mahesh K. Maskey, Kedar P. Baral, Rajani Shah, Bhagawan D. Shrestha, Janet Lang, Kenneth J. Rothman

**Affiliations:** 1 *Nepal Public Health Foundation, Kathmandu*; 2 *Patan Academy of Health Sciences, Nepal*; 3 *CTEVT, Bharatpur, Nepal*; 4 *Plan Nepal, Nepal*; 5 *Watson Institute for International Studies, Brown University, Providence RI, USA*; 6 *RTI Health Solutions, Research Triangle Park, NC & Boston University School of Public Health, USA*; * *Present address: Balsillie School of International Affairs, Waterloo, Ontario, Canada*

**Keywords:** Maternal mortality, millennium development goal, motherhood method, Nepal, sisterhood method

## Abstract

**Background & objectives:**

Measuring maternal mortality in developing countries poses a major challenge. In Nepal, vital registration is extremely deficient. Currently available methods to measure maternal mortality, such as the sisterhood method, pose problems with respect to validity, precision, cost and time. We conducted this field study to test a community-based method (the motherhood method), to measure maternal and child mortality in a developing country setting.

**Methods:**

Motherhood method was field tested to derive measures of maternal and child mortality at the district and sub-regional levels in Bara district, Nepal. Information on birth, death, risk factors and health outcomes was collected within a geographic area as in an unbiased census, but without visiting every household. The sources of information were a vaccination registry, focus group discussions with local health workers, and most importantly, interview in group setting with women who share social bonds formed by motherhood and aided by their peer memory. Such groups included all women who have given birth, including those whose babies died during the measurement period.

**Results:**

A total of 15161 births were elicited in the study period of two years. In the same period 49 maternal deaths, 713 infant deaths, 493 neonatal deaths and 679 perinatal deaths were also recorded. The maternal mortality ratio was 329 (95%CI:243-434)/100000 live birth, infant mortality rate was 48(44-51)/1000LB, neonatal mortality rate was 33(30-36)/1000LB, and perinatal mortality rate was 45(42-48)/1000 total birth.

**Interpretation & conclusions:**

The motherhood method estimated maternal, perinatal, neonatal and infant mortality rates and ratios. It has been field tested and validated against census data, and found to be efficient in terms of time and cost. Motherhood method can be applied in a time and cost-efficient manner to measure and monitor the progress in the reduction of maternal and child deaths. It can give current estimates of mortalities as well as averages over the past few years. It appears to be particularly well-suited to measuring and monitoring programmes in community and districts levels.

The current estimate of global maternal deaths is 342900^1^. Almost all of these occur in developing countries. Among the six countries accounting for more than 50 per cent of all maternal deaths, two South Asian countries, India and Pakistan occupy 1^st^ and 3^rd^ position^1^. Over the past decade, reduction in maternal deaths has attained a high priority in global health movements. The fifth Millennium Development Goal (MDG5) of improving maternal health has set a target of reducing the maternal mortality ratio by 75 per cent between 1990 and 2015^2^.

The most widely used measure of maternal mortality is the maternal mortality ratio, which is the ratio of the number of maternal deaths to the number of live births. It reflects (but is not identical to) the risk of maternal death once a woman has become pregnant. The 10^th^ Revision of the International Classification of Diseases (ICD-10) defined a maternal death as “the death of a woman while pregnant or within 42 days of termination of pregnancy, irrespective of the duration and site of the pregnancy, from any cause related to or aggravated by the pregnancy or its management but not from accidental or incidental causes”^3^. Maternal deaths are divided into direct and indirect obstetrical deaths. In practice the distinction between an accidental and incidental death or a direct and indirect death is problematic, and a precise cause of death may not be known despite knowledge of pregnancy. ICD-10 has, therefore, introduced an alternative definition of maternal death, the pregnancy related death, which emphasizes timing of death rather than the cause to which the death is attributed^3^. Many maternal mortality surveys, such as the sisterhood method^4^ typically measure pregnancy-related deaths as maternal deaths, since the cause of death is not elicited in such surveys.

The methods for measuring maternal mortality can be grouped into two categories: empirical and analytical.· A vital registration system, a facility-based health services records, and a census can be regarded as routine opportunity empirical measurement while population based surveys like sisterhood method and demographic surveillance systems can be considered as special opportunity empirical measurements^3^. Main analytical approaches are Birth and Death Record Linkage, Capture-recapture methods for correcting under-reporting of maternal deaths, and statistical modeling used by UN systems^3^. There could be composite approaches also such as Reproductive Age Mortality Study (RAMOS) and the Motherhood Method.

In most developing countries, vital registration of medically-certified births and deaths is non-existent or incomplete, and validity or feasibility of other purely records-based approaches is questionable. A reproductive age mortality study (RAMOS) uses multiple sources such as records from hospital, police, public-health department and vital data registries to identify and investigate the cause of deaths for each woman of reproductive age in a defined population. Interviews with household members and health care providers provide a basis to classify the deaths as maternal or otherwise. The RAMOS approach is considered to be the most complete estimation of maternal mortality, but it can be complex, because information regarding the number of births must come from separate sources^4^. RAMOS is generally less expensive than population based surveys or a complete census. All these types of studies are subject to under-ascertainment problems, despite their intensive use of resources^5^,^6^.

The sisterhood method is either indirect or direct. In recent years, the direct sisterhood method has been used for calculating the maternal mortality ratio (MMR) over a time reference of 0-6 or 0-13 years ration^7^. This approach uses 11 questions and more respondents. Surveyed participants provide information about their sisters – the number who reach adulthood, the number that have died, the age at death, the year in which the death occurred, and whether the death was during pregnancy, childbirth or shortly afterwards. Maternal mortality estimates from the sisterhood method have been useful in situations in which there is no other reliable measurement of the level of maternal mortality and limited resources hinder other approaches for measuring maternal deaths. But, it has many limitations. Although the direct method does not rely on assumptions about the patterns of fertility, it is less appropriate for settings with low fertility (total fertility rate <3) or a high level of migration; insufficient precision renders it less effective in comparing geographic areas (*i.e*. comparing sub-national estimates), studying trends, evaluating programme impact or allocating resources. Its use for measuring and monitoring the progress of intervention programmes aimed at reducing maternal mortality is particularly constrained owing to the fact that it cannot provide current estimates.

Some of these limitations can be overcome with the motherhood method. It is a direct technique for deriving local population-based estimates of maternal mortality, which can also be used as multi-stage cluster-sample estimates for larger populations. The method involves estimating the same information within a geographic area as would be collected in a census, but without visiting every household. It is a targeted census of births and deaths within a defined study period.

It is an evolutionary variant of the Participatory Community Survey method, which was developed to measure neonatal tetanus and the perinatal mortality rate in rural Nepal^8^,^9^. It shares features with the Boerma and Mati’s “networking” approach^10^ of eliciting maternal deaths and MIMF (Maternal death from Informants and Maternal death Follow-on review)^11^, which relies on interviews with individual mothers. It differs, however, in eliciting deaths through group discussion of listed mothers and community health care providers. It derives information about the numerator and denominator of the measure of interest directly from groups of women within the study area who share motherhood status by virtue of having given birth. To implement the method, the local health volunteers assist in facilitating group discussions related to maternal and child health. Information on total births and maternal death during pregnancy, childbirth or puerperium is elicited through immunization registries, group discussions (FGD), peer memory, memory aids and interview-based diagnosis (verbal autopsy). In this study we field tested this method to measure maternal and child mortality in a district in Nepal.

## Material & Methods

After pretesting the method in a small, relatively well-off community of about 8000 population^12^ which gave an estimate of MMR 140/100000, the method was tested in a larger sample of 15161 births in the Bara district of Nepal, where a child survival programme impact study was being conducted^13^. The sample size was expected to provide estimate of MMR within 30 per cent of margin of error. This study employed the pregnant women group (PWG) approach as a means to improve the maternal and child health status of the community^14^. The aim of the PWG approach is to empower the group in such a way that members are able to demand quality basic health services from governmental and non-governmental health care providers. The volunteers and participating women make all the decisions required to form and operate the group. The PWG comprised 7-15 pregnant women living in the same village or wards. They met once a month to discuss issues related to mother and child health. The female community health volunteers (FCHVs) facilitated these meetings.

Bara district is located in central terai plain of Nepal adjoining border with India. It has 98 village development committees and one municipality with one district hospital, three primary health care centers, 11 health posts and 84 sub-health posts. The total population projected for 2005 (based on 2001 census) was approximately 615,933. Of these, 130,578 were women of reproductive age (15-49 yr) and 98,241 were infants and children under five. It is a low human development index (HDI) district and has poor health indicators. The literacy gap between females and males was substantial, 14 and 42 per cent respectively. Muslims are second largest ethnic group in Bara^15^.

For the implementation of project, the district was divided into seven sectors. From each sector, seven Village Development Committees (VDCs), the administrative units having on an average six thousand population, were randomly selected making a total of 49 VDCs (50% of all VDCs in the Bara district, a total of 441 wards). Information regarding births, maternal death, infant death and PWG status over a study period of 2 yr from 17 July 2003 to 16 July 2005, was collected retrospectively from these VDCs in a survey period of approximately 12 wk. The data were checked every day for omissions and errors and corrected in the field by revisits when necessary. In this study, a sub-sample of 49 wards was randomly selected, one from each VDC, to conduct a census to validate the information obtained from the motherhood method.

Two days training was provided to supervisors and enumerators, and pre-testing and practice was done outside the study location to enable them to elicit required information from BCG and TT vaccination registries and from the group discussion. The study team prepared a list of mothers who had given birth in the study period by collecting information from local BCG and TT vaccination registries. BCG vaccination is given in the first week of birth to immunize against tuberculosis. In rural areas the vaccination may be delayed by a month or more, so some babies who die early in the neonatal period may not be listed in the registry. Because hospitals may vaccinate babies with BCG without recording the information in the local BCG registry, and because some deliveries take place at the homes of relatives, local BCG registries may have incomplete information about local births. These limitations of BCG registries were partially compensated for by augmenting the list from TT vaccination registries.

Mothers who had taken even a single dose of TT in pregnancy were included in the list because the objective was to identify the pregnancy status of the study subjects. To capture most of the births that would fall within the study period, TT vaccination information was collected from 17 April 2003 through 16 July 2005, three months before the study period. Mothers who received in these three months their first dose of TT while they were in the last trimester of pregnancy were likely to complete their pregnancy at the beginning of the study period, whereas those receiving vaccine during the first or second trimester were likely to complete their pregnancy later during the study period.

The augmented list was given to the female community health volunteers to pass on to the mothers. The study objectives were explained to each mother, and those who gave consent to participate were asked to assemble at a fixed time and place for the group discussion. The typical group comprised 10-15 mothers and the local health workers. The focus group discussion with the mothers and local health workers emphasized the pregnancy outcomes of these mothers and checked whether they were within the study period. Deliveries outside the study period were excluded from the list of counted pregnancies.

At the group discussion, the mothers on the list were asked the date of birth of their baby or babies. Most could recall the exact birth date, although some could remember only month and year. The listed information was considered correct if mother’s information corroborated it. The group discussion also elicited information about maternal deaths, infant deaths, stillbirths and abortions. Some mothers had better recall about these events than others. Any conflict in group’s opinion was resolved by interviewing the woman in question or another household member. Those who could not come to the group discussion were visited in their own household. For mothers who had died within the study period, a close relative (mother, mother-in law, or husband) was interviewed to ascertain whether the death was a maternal death.

The results were validated by conducting a census of remaining households not included in the list of study births. The census data were used to estimate the sensitivity and specificity of the method for ascertaining births and deaths. Overall it took 6 wk to collect data from 49 VDCs, including FGD and census in 49 wards. There were seven groups, each with four data collectors with 3 enumerators and one supervisor. On an average one group took five days to cover one VDC.

The total cost of the evaluation was $ 10,896. The allowance for FCHV was $ 905 (Rs 2.05 × 49vdc × 9 wards × 1 day). It was found that doing a census was 10 times more costly than collecting data from motherhood method, (per unit cost $ 50.5 and $ 4.4).

## Results

Of the 15,161 births (14,916 live births, 245 still births), there were 97 twin births, one triplet birth and 128 births from mothers who had given birth previously during the study period. Seven hundred thirteen babies died in infancy, of whom 493 (69%) died in the neonatal period and 434 (61%) in the early neonatal period. The number of maternal deaths was 49. Table I presents the distribution of births and deaths in all the 441 wards of the sampled 49 VDCs, the validation data of the census and motherhood method in 49 wards and the findings in the remaining 392 wards. In the 49 wards, the Census recorded 1,995 live births, 25 stillbirths, 93 infant deaths, 77 perinatal deaths and 6 maternal deaths. The motherhood method elicited 1,990 live births, 25 stillbirths, 93 infant deaths, 77 perinatal deaths and 6 maternal deaths in the same wards. The only discrepancy was five live births recorded from the census that were missed by the motherhood method. Among the 392 remaining wards, there were 12,921 live births, 220 still births, 620 infant deaths and 43 maternal deaths during the same period.

**Table I:** Total births and deaths by study groups, Bara district

Category	Total wards surveyed (441) of 49 VDC				Total	PWG	Non-PWG
	392 wards [motherhood method (MM)]	49 wards (census)					
		Census	MM	Under-estimation (%)			
Live birth	12,921	1,995	1,990	0.25	14,916	4,334	10,582
Still birth	220	25	25		245	41	204
Total birth	13,141	2020	2015	0.25	15,161	4,375	10,786
Maternal death	43	6	6		49	9	40
Infant death	620	93	93		713	108	605
Neonatal death	435	58	58		493	81	412
Early neonatal death	382	52	52		434	72	362
Perinatal death	602	77	77		679	113	566

PWG, pregnant women group; VDC, village development committee

**Table II:** Mortality indices of Bara district compared with national estimates

Mortality rates	PWG	Non-PWG	Total (95% CI)	National average
Maternal mortality ratio/100,000 live birth	9/4334=208	40/10582=378	329 (243- 434)	281*
Infant mortality rate/1000 live birth	108/4334=25	605/10582=57	48 (44 - 51)	48*
Neonatal mortality rate/ 1000 live birth	81/4334=19	412/10582=39	33 (30 - 36)	33*
Early neonatal mortality rate/1000 live birth	72/4334=17	362/10582=34	29 (26 - 32)	
Perinatal mortality rate/1000 total birth	113/4375=26	566/10786=52	45 (42 - 48)	45*
Still birth rate/1000 total birth	41/4375=9.4	204/10786=19	16 (14 -18)	8.5**

* Nepal Demographic and Health Survey 2006^18^

** Estimate of Kathmandu population^19^; PWG, pregnant women group

Mortality rates were computed for mothers who were and were not part of the PWG, with 95 per cent confidence intervals (Table II). Overall, the maternal mortality ratio (MMR) was 329/100000 live births (LB), the infant mortality rate (IMR) was 48/1000 LB, the neonatal mortality rate (NMR) was 33/1000 LB, and the early neonatal mortality rate was 29/1000 LB. The perinatal mortality rate (PMR) and stillbirth rate (SBR) were calculated with total births in the denominator and were 45/1000 TB and 16/1000 TB respectively.

The results compared well with national data. A comparison with the census results in 49 wards showed 100 per cent agreement with MM in detecting maternal and child deaths. There was about a 0.25 per cent under-reporting of births. The maternal, infant, neonatal and perinatal indicators in PWG women were lower than the non-PWG women and the national statistics.

## Discussion

Field-testing of the motherhood method in a district with a population of about 600,000 demonstrated that maternal mortality can be directly measured if the BCG and TT vaccination registers are in place and local health workers or volunteers and the mothers themselves in the wards are properly mobilized and supervised for data collection. The possibility of missing maternal deaths in early pregnancy and those related to abortion being reported as non maternal deaths cannot be ruled out, but such under-reporting can be reduced by collecting the information about all female deaths and then using a careful verbal autopsy in the group settings. Proper motivation of community key informants, health volunteers, and mobilizers is crucial for the accuracy of data.

The findings show that the motherhood method can be applied in a time and cost-efficient manner to measure and monitor the progress in the reduction of maternal and child deaths. It approximated census-based measurement while at the same time remaining relatively immune to the problem of omission and misclassification of numerator and denominators in census studies. It can give current estimates of maternal mortality as well as averages over the past few years. It appears to be particularly well-suited in measuring and monitoring programmes in sub-national regions and districts.

The mix of ‘outsider’ field assistants and ‘insider’ local health volunteers used appears to have been able to keep information errors down, thus improving accuracy of information and increasing time efficiency of interview. It appeared that the group discussion effectively counteracted the disinclination of mothers to talk about the death of their child, and enhanced collective memory for recalling details related to maternal and child mortality. Where confidentiality was indicated, interviews were conducted with mothers or family members in absence of local health workers.

The motherhood method also appears to be robust regarding problems induced by migration. The group discussion could elicit which mothers migrated to the village to live or came to their mother’s home for delivery.

The method has its limitations. It requires proper training of field assistants to moderate the group discussion among mothers and health volunteers. Motivation of key community informants and health volunteers is crucial to the accuracy of data, and mothers need to be aware of the need for accuracy. Although the method is efficient, the effort in collecting data depends on the duration of the study period, the longer the study period, the greater the potential for inaccurate recall. It is likely that some maternal deaths related to ‘hidden pregnancy’, particularly among teens be missed. Reporting of maternal deaths in early pregnancy and those related to abortion as non-maternal deaths may occur. Collecting information about all female deaths and the careful application of verbal autopsies in the group setting may reduce such misclassification. The method would need further adaptation to measure births and deaths in urban areas.

The International Conference on Population and Development +5 program of Action (1999) “calls upon United Nations and donors to support developing countries in undertaking census and surveys and to develop innovative and cost effective solution for improving estimates of maternal mortality”^16^. For economically poor countries, measuring maternal mortality has been viewed as “notoriously difficult and complex” and characterized as nearly hopeless by agencies such as the WHO, who maintained that “the problem of measuring maternal mortality is most acute precisely where it is least likely to be accurately measured^17^”. Our experience in Nepal needs more refinement, and validation at the national level. The present results provide the ground to take initiatives for development and validation of similar methods, and ultimately for the development of a commonly agreed upon methodology in other developing countries, especially in South Asia.
